# Effect on Blood Pressure of Daily Lemon Ingestion and Walking

**DOI:** 10.1155/2014/912684

**Published:** 2014-04-10

**Authors:** Yoji Kato, Tokio Domoto, Masanori Hiramitsu, Takao Katagiri, Kimiko Sato, Yukiko Miyake, Satomi Aoi, Katsuhide Ishihara, Hiromi Ikeda, Namiko Umei, Atsusi Takigawa, Toshihide Harada

**Affiliations:** ^1^Faculty of Health and Welfare, Prefectural University of Hiroshima, Mihara, Hiroshima 723-0053, Japan; ^2^Central Laboratory, Research & Development Division, Pokka Sapporo Food and Beverage Ltd., Kitanagoya, Aichi 150-0013, Japan

## Abstract

*Background*. Recent studies suggest that the daily intake of lemon (*Citrus limon*) has a good effect on health, but this has not been confirmed in humans. In our previous studies, it was observed that people who are conscious of their health performed more lemon intake and exercise. An analysis that took this into account was required. * Methodology*. For 101 middle-aged women in an island area in Hiroshima, Japan, a record of lemon ingestion efforts and the number of steps walked was carried out for five months. The change rates (Δ%) of the physical measurements, blood test, blood pressure, and pulse wave measured value during the observation period were calculated, and correlations with lemon intake and the number of steps walked were considered. As a result, it was suggested that daily lemon intake and walking are effective for high blood pressure because both showed significant negative correlation to systolic blood pressure Δ%. *Conclusions*. As a result of multiple linear regression analysis, it was possible that lemon ingestion is involved more greatly with the blood citric acid concentration Δ% and the number of steps with blood pressure Δ%, and it was surmised that the number of steps and lemon ingestion are related to blood pressure improvement by different action mechanisms.

## 1. Introduction

It has been reported that food materials such as tea catechins, cacao lignin, and wheat bran hemicellulose had a suppressive effect on blood pressure in spontaneously hypertensive rats (SHR) [1–3].

Several studies highlighted lemon as an important health-promoting fruit rich in phenolic compounds as well as vitamins, minerals, dietary fibers, essential oils, and carotenoids [4].

The suppressive effect of lemon juice and its crude flavonoid on blood pressure has been reported in SHR [5]. The water extract of lemon peels also had a suppressive effect on blood pressure in SHR [6, 7]. Recently, we investigated the effect of daily lemon intake on parameters related to metabolic syndrome in healthy women and showed that the amount of lemon intake had a significant negative correlation with systolic blood pressure [8].

On the other hand, it has been well known that daily walking improves the blood pressure in humans [9].

In this study, we examined the contribution of daily lemon intake and daily walking to blood pressure and related parameters in middle-aged and older women in their daily life.

## 2. Materials and Methods

### 2.1. Subjects

A hundred elevenwomen aged 33 to 77 (mean ± SD: 60.9 ± 8.0) who resided on islands in the Seto Inland Sea of Hiroshima Prefecture were included in this study. This area is known for lemon production in Japan. In September 2011 and in March 2012, we conducted a survey of physical measurements and biochemical and serological parameters. Of these 111 women, 10 women who were treated for hypertension, diabetes, or hypercholesterolemia therapy were excluded in data analysis. In the period between the first survey (September 2011) and the second survey (March 2012), they were requested to wear the record-type pedometer (Lifecorder EX, Suzuken Co. LTD., Japan) and to self-record their daily lemon intake. The lemon ingestion method was not specified.

### 2.2. Methods

The physical characteristics were measured with a Body Composition Analyzer (BC-118D, Tanita Co., Ltd., Japan). Blood pressure and pulse wave velocity were measured with inspection apparatus (Form BP-203 RPE, Omron Co., Ltd., Japan). Blood samples were sent to a privately owned clinical laboratory for biochemical and serological testing. There were 32 physical characteristics and biochemical and serological measurements examined, as shown in Table 1.

### 2.3. Data Analysis and Statistics

The amount of lemon intake was presented as an average number of lemons per day. Movement was expressed by the average number of steps per day according to the pedometer (Lifecorder EX, Suzuken Co., Ltd., Japan). The value of physical characteristics and biochemical and serological examinations was shown as the change rate (Δ%) of the value measured in March against the value measured in September (100%). The means and standard errors of Δ% were calculated. The data were compared between average values recorded in September and in March using Student's* t*-test.

The correlations between the lemon intake number per day, daily number of steps, and the change rate of each physical characteristic and biochemical and serological data were tested using Pearson's correlation coefficient.

Multiple regression analysis was performed using the number of steps per day and lemon intake as the independent variable and the change rates of systolic blood pressure and blood concentration of citric acid as the dependent variable.

All statistical analyses were performed using statistical software (SPSS version 17.0, SPSS Inc., USA) on personal computers, and *P* < 0.05 was considered as significant.

### 2.4. Ethics Statement

Based on Declaration of Helsinki, we explained the meaning and the methods of research to all the subjects and obtained written consent. The plan of research was recognized by the Research Ethics Committee of the Faculty of Health and Welfare of the Prefectural University of Hiroshima (M11-0024).

## 3. Results

The lemon intake per day during the entire study was 0.47 ± 0.47 (mean ± standard deviation), but there were particularly many omissions and deficiencies in the record of the early days of the investigation because it was self-recorded by the subjects. In February, which is the last part of the investigation, a complete record was 0.43 ± 0.49. Since the lemon intake of February and the whole period had strong correlation (correlation coefficient 0.947, *P* < 0.000), the data in February was used for analysis afterward. The number of steps per day throughout the entire period of investigation was 6947.74 ± 2187.20.

Table 1 shows the averages and standard error of the Δ% of measurement items. Obesity-related indexes, such as the weight, blood fat, and insulin, significantly increased. In addition, the iron-related indexes, such as a red blood cell and the hemoglobin and citric acid, increased.

**Figure 1:**
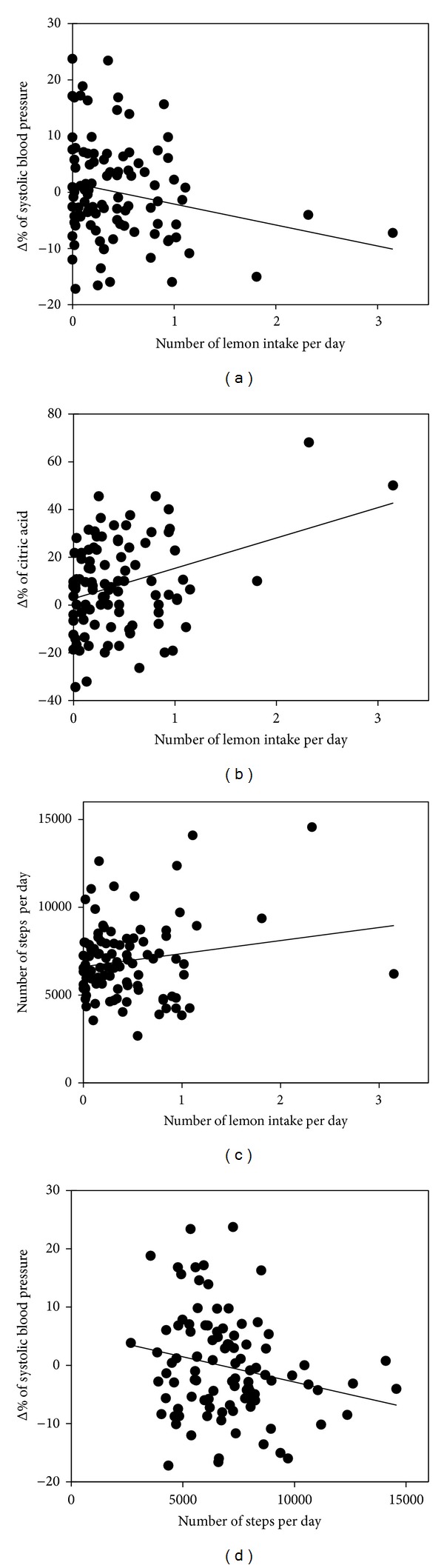
Scatter plot of lemon intake, number of steps, and Δ% of systolic blood pressure and citric acid. Linear approximations were shown in each graph as line ((a): *R* ^2^ = 0.041, (b): *R* ^2^ = 0.117, (c): *R* ^2^ = 0.027, and (d): *R* ^2^ = 0.046).

The number of lemon intake per day correlated negatively with the Δ% of systolic blood pressure and red blood cells and positively with the Δ% of blood citric acid concentration. Similarly, the number of steps per day correlated negatively with the Δ% of systolic blood pressure and positively with blood citric acid concentration. All these correlations were significant. On the other hand, the Δ% of blood citric acid concentration showed negative and significant correlation with the Δ% of systolic blood pressure and pulse pressure and significantly positive correlation with red blood cells (Figure 1, Table 2). 

Table 3 shows the results of multiple regression analysis. Lemon intake and number of steps walked were identified as the significant factors associated negatively with systolic blood pressure and the number of steps was more effective than the lemon intake. On the other hand, lemon intake and number of steps were identified as the significant factors associated positively with Δ% of blood citric acid concentration, and the lemon intake was more effective than the number of steps (Table 3).

## 4. Discussion

Citrus fruits are often reported to be foods which are beneficial for health [10–12]. The lemon, a representative citrus fruit, includes bioactive components such as citric acid, polyphenol, and ascorbic acid, and it is reported that there are various health benefits [4]. Previously, we reported a systolic blood pressure-lowering effect by lemon ingestion in Japanese people [8]. In the present study, keeping in mind the effect due to the motion of the subject, we confirm the effect of lemon intake.

During the five months obesity-related indicators of the subjects increased (Table 1). According to animal and* in vitro* experiments, it was reported that the lemon has effective ingredients for lipid metabolism and obesity [13–15]. But this effect could not be confirmed in this investigation. This is thought to be due to the seasonal cycle of the subjects. It was reported in at least Japanese people that the percentage of body fat increases in the winter season [16]. Furthermore, subjects of this investigation were older and middle-aged farm women. Since the survey period, from September through March, included the agricultural off-season, it is inferred that exercise time through work was significantly reduced. Therefore, it is thought that skeletal muscle masses decreased and the fat mass increased. From the fact that brachial-ankle pulse wave velocity increased and diastolic blood pressure significantly decreased, it is expected that the angiosclerosis in subjects was advancing, probably due to aging. The suppression of the increase in systolic blood pressure in this study may be due to enhanced metabolism such as urination.

The amount of lemon intake of the subjects prior to the survey period is not clear as a number. According to the prior interview, although subjects were from lemon-production farmhouses, they did not usually intentionally eat lemons. Rather, like the average Japanese, most only used them for cooking occasionally. The duty of the ingestion record of lemons and wearing of a pedometer was imposed during the survey period, and it is expected that this became the motivation to health consciousness. The increase in a lemon intake during the survey period is indirectly shown in the increase in the citrate in serum (Table 1).

The blood pressures, especially systolic pressure, showed significant negative correlation to the lemon intake and the number of steps. On the other hand, the number of steps and lemon intake tend to be in correlation (Figure 1, Table 2). It is guessed that subjects having high health consciousness ate lemons often and moved often. This means that the results shown in Table 2 are not simply dependent on the intake of lemon.

In this study, we focus on the citric acid among the components contained in the lemon. Citric acid Δ% varied strongly by the lemon intake rather than by the number of steps in a result of multiple linear regression analysis (Table 3). Therefore, it is suggested that this blood citric acid is due to the lemon.

Though the time-to-maximum blood concentration of citric acid is 0.8 hr and the half-life in blood is 2.3 hr, it is interesting that citric acid Δ% had a strong association with lemon intake and steps walked (Figure 1, Table 2) [17, 18]. That there was a cumulative effect with repeated administration is to be inferred.

Citric acid Δ% correlated with Δ% of systolic blood pressure, pulse pressure, and red blood cell count (Table 2). It is not out of the realm of analogy at all about the mechanism of action of citric acid in the present study. One of the reported properties of citric acid is that it generates calcium citrate from inorganic salts, such as calcium phosphate or calcium carbonate, to promote absorption from the intestinal tract [19–21]. In addition, there is the report that the citric acid of citrus fruits promoted absorption of calcium and magnesium in food [22]. There is a possibility that calcium and magnesium which were absorbed efficiently are participating in blood pressure [23]. Also apart from the effect of citric acid in food, it is necessary to consider the effects of intracellular citric acid or the citrate in blood. The citric acid Δ% showed a positive correlation with the number of red blood cells Δ% but showed a negative correlation with lemon intake (Table 2). The mechanism is not clear, but this implies that the citric acid is involved in the blood composition. The lemon ingestion method in this research was not specified. It is believed that most subjects ingested only juice, but the ingestion of peels was not prohibited. Flavonoids of lemon are mainly contained in the peel and there are reports that the ingredient acts on the reduction in systolic blood pressure [4, 15]. It is possible that actions by ingredients other than citrate are also involved intricately.

The number of steps showed a negative correlation with systolic blood pressure Δ% and a positive correlation with citric acid Δ% (Table 2). Blood pressure improvement through walking has been reported [9]. Even in the results of heavy regression analysis of the present study, it has been shown that systolic blood pressure Δ% has been affected more strongly by the walking than by lemon ingestion (Table 3). Because of the amount of walking having been proportional to the lemon intake, but not having been related to the number of red blood cells Δ%, and the result of the multiple regression analysis, it is believed that the amount of walking is not applied to the citric acid Δ% directly.

These results suggest that the walking and lemon ingestion have the effect of lowering systolic blood pressure by, respectively, different action mechanisms. There may be an additive or synergistic effect in movement and lemon ingestion.

The habits of lemon ingestion before the investigation are not taken into consideration in this research. The method of lemon intake is also not clear, or how the peel was included. There is a need to examine blood metal ions as a clue to investigate the mechanism of action in the body of lemon components. A longer experimental period is required to study the effect on pulse wave velocity and diastolic blood pressure related to atherosclerosis [24, 25]. Noting these future studies, using a placebo in consideration of the health awareness of the subject, a long-term study of more than one year or a short-term study with less seasonal variation is desired.

## Figures and Tables

**Table 1:** Data of physical characteristics, blood vessel related values, and blood test.

	Values on September mean ± SD	Values on March mean ± SD	*P* values on between September and March	Change rate (Δ%) mean ± SE
Physical measurement				
Body weight (kg)	54.8 ± 8.3	55.4 ± 8.4	0.001	1.03 ± 0.29
Body mass index (kg/m^2^)	23.3 ± 3.1	23.5 ± 3.1	0.005	1.07 ± 0.37
Body fat ratio (%)	33.1 ± 6.1	35.4 ± 6.0	<0.000	7.68 ± 1.09
Lean body mass (kg)	36.3 ± 3.4	35.4 ± 3.4	<0.000	−2.43 ± 0.24
Blood pressure, arteriosclerosis relation				
Systolic blood pressure (mmHg)	130.0 ± 16.3	130.0 ± 18.5	0.871	0.02 ± 0.89
Diastolic blood pressure (mmHg)	75.3 ± 9.2	73.4 ± 10.9	0.014	−2.37 ± 1.02
Pulse pressure (mmHg)	54.5 ± 9.8	55.9 ± 10.3	0.058	3.53 ± 1.40
Ankle Brachial Pressure Index	1.1 ± 0.1	1.1 ± 0.1	0.334	1.13 ± 0.80
Brachial-ankle pulse wave velocity (cm/sec)	1484.6 ± 286.4	1555.5 ± 322.4	0.058	5.08 ± 1.09
Blood test				
White blood cell (/*μ*L)	5649.5 ± 1491.7	5922.8 ± 1583.5	0.013	6.49 ± 2.05
Red blood cell (∗10^4^/*μ*L)	424.1 ± 29.5	437.9 ± 321.4	<0.000	3.30 ± 0.41
Hemoglobin (g/dL)	12.8 ± 1.0	13.1 ± 1.0	<0.000	3.04 ± 0.57
Hematocrit (%)	40.0 ± 2.8	41.1 ± 2.8	<0.000	3.73 ± 0.54
Platelet (∗10^4^/*μ*L)	24.5 ± 5.3	25.4 ± 5.7	0.005	4.24 ± 1.31
Mean corpuscular Volume (fL)	93.7 ± 4.3	93.9 ± 4.0	0.297	0.42 ± 0.36
Mean corpuscular hemoglobin (pg)	30.1 ± 1.7	30.0 ± 1.56	0.317	−0.24 ± 0.42
Mean corpuscular hemoglobin concentration (%)	32.2 ± 0.96	32.0 ± 0.77	0.019	−0.63 ± 0.29
Hemoglobin A1c (%)	5.4 ± 0.4	5.3 ± 0.43	0.018	−0.72 ± 0.28
Serum iron (*μ*g/dL)	86.8 ± 26.7	92.9 ± 30.0	0.032	12.99 ± 4.20
Unsaturated iron binding capacity (*μ*g/dL)	225.1 ± 54.7	222.2 ± 47.1	0.384	0.68 ± 1.66
Reticulocyte (‰)	11.2 ± 3.4	10.4 ± 2.8	0.002	−3.99 ± 2.22
Total cholesterol (mg/dL)	208.0 ± 33.1	214.6 ± 33.9	0.003	3.65 ± 2.12
Neutral fat (mg/dL)	139.2 ± 79.1	139.5 ± 69.7	0.979	11.89 ± 5.05
High density lipoprotein cholesterol (mg/dL)	63.4 ± 15.4	64.5 ± 14.5	0.178	2.69 ± 1.28
Low density lipoprotein cholesterol (mg/dL)	119.5 ± 28.1	124.1 ± 30.0	0.012	4.74 ± 1.43
Uric acid (mg/dL)	4.37 ± 0.9	4.30 ± 0.9	0.245	−0.83 ± 1.31
Insulin (*μ*U/mL)	17.1 ± 16.1	21.8 ± 19.8	0.012	95.45 ± 20.69
Cortisol (*μ*g/dL)	50.4 ± 19.3	60.8 ± 22.7	<0.000	39.37 ± 7.70
Adiponectin (*μ*g/mL)	9862.0 ± 5275.5	12418.0 ± 6636.6	<0.000	37.20 ± 7.42
Leptin (ng/mL)	6.4 ± 5.1	8.7 ± 5.3	<0.000	96.76 ± 21.08
Citric acid (mg/dL)	2.7 ± 0.4	2.9 ± 0.6	<0.000	8.20 ± 1.90
Renin (pg/mL)	565.0 ± 317.8	538.8 ± 318.9	0.263	3.43 ± 4.80

**Table 2:** Pearson coefficient of correlation of significantly different or tendency items.

	Lemon intake per day		Number of steps per day		Citric acid (Δ%)	
	Correlation coefficient	*P* value	Correlation coefficient	*P* value	Correlation coefficient	*P* value
Number of steps per day	0.167	0.099				
Citric acid (Δ%)	0.343	<0.000	0.213	0.042		
Systolic blood pressure (Δ%)	−0.204	0.040	−0.222	0.034	−0.242	0.015
Diastolic blood pressure (Δ%)	−0.171	0.088	−0.151	0.152	−0.058	0.562
Pulse pressure (Δ%)	−0.181	0.069	−0.188	0.074	−0.318	0.001
Red blood cell (Δ%)	−0.222	0.025	0.098	0.358	0.215	0.031

**Table 3:** Multiple regression analysis for the relationship between change rate of systolic blood pressure and blood citric acid concentration as dependent variables versus number of lemon intake and steps per day as independent variables.

	Systolic blood pressure (Δ%) (*P* = 0.32)		Citric acid (Δ%) (*P* = 0.01)	
	*β* coefficient	*P* value	*β* coefficient	*P* value
Number of steps per day	−0.190	0.060	0.154	0.113
Lemon intake per day	−0.153	0.128	0.302	0.002
